# 

**Published:** 1853-10

**Authors:** 


### RECEIPTS

### FOR THE JOURNAL DURING THE MONTH OF SEPTEMBER.

ILLINOIS.

| | |
|---|---|
| Dr. J. B. Albright, | $2 00 |
| " P. E. Rosenberg, | 2 00 |
| " S. M. Reynolds, | 4 00 |
| " Julius P. Anthony, | 1 00 |
| " J. Blount, | 2 00 |
| " Lynn | 2 00 |
| " E. M'Arthur | 2 00 |
| " D. Quinlan, | 2 00 |
| " Boon, | 4 00 |
| " Morfit, | 2 00 |
| " Palmer, | 4 00 |
| " Hiram Smith, | 2 00 |
| " Jas. L. Cram, | 2 00 |
| " J. N. Banks, | 4 00 |
| " Milton Bacon, | 6 00 |
| " D. Ray. | 2 00 |
| " P. C. Morier, | 2 00 |
| " F. Scammon, | 4 00 |
| " V. L Hurlbut, | 2 00 |
| " Henrotin, | 2 00 |

WISCONSIN.

| | |
|---|---|
| Dr. Cutting Marsh, | 2 00 |
| " W. P. M'Allister, | 2 00 |
| " C. C. Warner, | 4 00 |

INDIANA.

| | |
|---|---|
| Dr. A. S. M'Curdy, | 2 00 |
| " M. F. Miller, | 2 00 |
| " S. R. Youngman, | 2 00 |
| " J. Davenport, | 2 00 |
| " Hiram Duncan, | 4 00 |
| " Joshuah D. Gray, | 4 00 |
| " Joseph P. Russell, | 4 00 |
| " J. B. Buchtel, | 2 00 |
| " F. Sampson, | 2 00 |

IOWA.

| | |
|---|---|
| D.r H. L. Whiteman, | 4 00 |
| " J. Diron, | 2 00 |

MISSOURI.

| | |
|---|---|
| Dr. S. D. Golder, | 2 00 |

MICHIGAN.

| | |
|---|---|
| Dr. A De Armand, | 5 00 |
| " Leander Osborn, | 2 00 |
| " C. B. Goodrich, | 2 00 |
| " J. W Hall, | 2 00 |
| " R. Stephenson. | 2 00 |

MINNESOTA TERRITORY.

| | |
|---|---|
| Dr. A. E. Ames, | 2 00 |
| " J. H. Murphy, | 2 00 |

